# Beyond Skin Deep: case-based online modules to teach multidisciplinary care in dermatology among clerkship students

**DOI:** 10.1186/s12909-023-04072-z

**Published:** 2023-02-04

**Authors:** Chaocheng Liu, Megan Chan, Vivienne Beard, Pamela Mathura, Marlene Dytoc

**Affiliations:** 1 Department of Dermatology and Skin Science, University of British Columbia, 835 West 10th Ave, 3rd Fl, Vancouver, BC V5Z 4E8 Canada; 2 Faculty of Medicine, University of British Columbia, Vancouver, BC Canada; 3 Alberta Health Services and Department of Medicine, University of Alberta, Edmonton, AB Canada; 4 Division of Dermatology, Faculty of Medicine and Dentistry, University of Alberta, Edmonton, AB Canada

**Keywords:** Case-based learning, Multidisciplinary care in dermatology, Undergraduate medical education, Online learning

## Abstract

**Background:**

Canadian medical schools offer limited clinical dermatology training. In addition, there is a lack of educational resources that are designed specifically for clerkship students that focus on the multidisciplinary nature of dermatology.

**Objectives:**

After developing case-based educational resources to address the lack of clinical exposure and learning of multidisciplinary care in dermatology, this study aimed to evaluate the educational intervention and gather feedback for future module development.

**Methods:**

Ten online interactive dermatology case-based modules involving 14 other disciplines were created. Medical students (*n* = 89) from two Canadian schools were surveyed regarding perceptions of the existing dermatology curriculum. Among 89 students, 46 voluntarily completed the modules, and a survey (a five-point Likert scale ratings) including narrative feedback was provided to determine an improvement in dermatology knowledge and understanding of multidisciplinary care.

**Results:**

Among 89 surveyed students, only 17.1% agreed that their pre-clerkship dermatology education was sufficient and 10.2% felt comfortable managing patients with skin conditions in a clinical setting. Among 46 students, 95.7% of students agreed that the modules fit their learning style (4.17 ± 0.73 on Likert scale) with positive narrative feedback. 91.3% agreed or strongly agreed that the modules enhanced their dermatology knowledge (4.26 ± 0.61). 79.6% of students agreed that the modules helped with understanding the multidisciplinary nature of dermatological cases (3.98 ± 0.81). Student comfort to manage skin conditions increased 7.7 times from 10.2% to 78.3% post-module.

**Conclusions:**

Clerkship students had limited knowledge of dermatologic conditions; the case-based modules were able to successfully address these deficits and assist students in understanding the multidisciplinary nature of dermatology.

**Supplementary Information:**

The online version contains supplementary material available at 10.1186/s12909-023-04072-z.

## Introduction

Skin conditions are a leading cause of disease burden worldwide in terms of prevalence and morbidity [1]. Skin presentations manifest throughout various aspects of medicine. Both family physicians and specialists in many fields of medicine are routinely met with skin conditions or dermatologic manifestations of various diseases [2–4]. Given the high prevalence of skin conditions, understanding foundational dermatology is crucial for all graduating medical students. Despite this, teaching medical students who have no intention of specializing in dermatology the relevance of dermatology to their specialties of interests is currently not cited as a mandate in the Canadian Professors of Dermatology's National Dermatology Core Curriculum and Competencies [5]. In addition, Canadian national surveys from 1983 to 2018 consistently highlighted the disproportionately low level of dermatology teaching in relation to the amount of skin disease managed by physicians in practice [6, 7]. In a survey for all dermatology directors at each Canadian medical school that was conducted in 2018, only 29% of them thought the dermatology education provided at their medical school was adequate [6]. The hours of teaching on dermatology were limited for the majority of the schools and the lowest number reported is 3 h [6]. Of all teaching hours, 75% is offered during the preclerkship stage, and only 3 medical school offered mandatory clinical experience in dermatology [6]. In Canada, there appears a lack of dermatology faculty, as well as insufficient time and resource allocation for dermatology in the medical doctoral (MD) curriculum [6].

Clerkship provides an experiential opportunity for dermatology education and exposure in clinical multidisciplinary settings. First, the main issue with the undergraduate dermatology teaching is the lack of clinical exposure to dermatology. In addition, in Canada, a clerkship rotation in dermatology is not always mandated [6]. The lack of dermatology education for medical students is not an issue limited to Canada. In the United States, the most recent survey of all 137 medical schools in 2019 revealed that only 12% of them had a course dedicated solely to dermatology in their preclerkship curricula and 1% had a required third-year clinical rotation in dermatology ranging from one to four weeks [7]. Reported challenges included lack of dermatology departments to assist, difficulty incorporating substantial dermatologic education into existing courses, and limited time in the schedule [7]. There is a similar need to improve the standards of dermatology teaching, learning, and assessment for Australian medical schools [8]. Although certain core learning outcomes were addressed in their medical school curricula, there is a lack of education on common problems like dermatophyte infections, drug reactions, and dermatologic emergencies [8]. In the United Kingdom, there is no mandatory dermatological component in medical school curricula despite its high prevalence of skin diseases (12.4%) in the primary setting [9].

Based on the authors’ teaching experiences, there is a gap in study resources available to medical students, specifically materials that focus on teaching the multi-disciplinary nature of dermatology. In addition, due to the lack of clinical exposure, students may fail to have a full understanding of the relevance of dermatology to other specialties or the clinical practice of dermatology. Students often do not have the opportunity to see the interdisciplinary aspects of dermatology, for examples, in psychodermatology, hematology-dermatology or rheumatology-dermatology combined clinics. Given these challenges, developing educational interventions to increase clinical exposure in dermatology for clerkship students is a critical part of ensuring that Canadian medical schools graduate well-rounded physicians with the capability to assess and manage skin manifestations of various diseases and understand the importance of multidisciplinary care in dermatology when taking care of patients with complex skin conditions.

After creating an educational resource guided by Thomas and Kern’s framework for clerkship students to address the lack of clinical exposure and learning of multidisciplinary care in dermatology, this study aimed to evaluate the educational intervention and gather feedback for development of future interventions on this topic [10].

## Materials and methods

### Guiding framework

The development, implementation, and evaluation of the educational intervention were guided by the six-step approach of Thomas and Kern’s Curriculum Development for Medical Education. Thomas and Kern’s Curriculum Development for Medication Education is a frequently used approach for medical educators as a framework to develop educational interventions when there is a learning gap [10].

### Study setting

Our local institutions (University of Alberta and University of British Columbia) are two Canadian medical schools that both provide a 4-year MD curriculum and have a one-week designated time for dermatology teaching in preclerkship years which is standard for Canadian medical school training.

### Targeted needs assessment

To better understand the issue of lack of clinical exposure and learning of multidisciplinary care in dermatology at local institutions, we designed a needs assessment survey to gather demographic information, educational resources used by students to study dermatology, perception of pre-clerkship dermatology curricula, and understanding of the multidisciplinary nature of dermatology among clerkship students (i.e., third- and fourth-year medical students on clinical rotations). The survey questions were developed based on the literature review of other educational interventions in the areas of dermatology and multi-disciplinary care as well as reviewing the learning objectives for dermatology in the existing MD curriculum. The survey development team included a practicing dermatologist, a dermatology resident, and medical students who all provided feedback regarding question content, format, and wording. The survey content was then reviewed by five clerkship medical students and minor revisions were made before distribution to participants.

### Goals/objectives & educational intervention (i.e., Module creation)

After determining the learning gap identified with the needs assessment survey, our team consisting of an academic dermatologist, a dermatology resident, a quality improvement specialist, and medical students was formed with the goal of creating educational resources for clerkship medical students, to enhance dermatology knowledge and improve the understanding of the multidisciplinary nature of dermatology. The educational intervention is named “Beyond Skin Deep Module” which composed of a set of 10 interactive cases that are designed for medical students to use as learning resources (Table 1). First, we conducted a literature review to assess the format and content of educational resources to enhance dermatology knowledge among medical students. The search was conducted on MEDLINE, EMBASE, and PUBMED using key words of “medical school”, “medical student”, “dermatology”, and “medical education”. All articles were screened by the authors to identify educational relevant intervention of any format. In addition, we reviewed recommended curricular and non-curricular online dermatology and non-dermatology learning resources amongst clerkship students. Based on our review, we identified educational interventions that improved student performance. We then summarized the key features of identified interventions that included case based, question–answer format along with detailed explanation, concise take-home messages, and cases of diverse social backgrounds. Based on these key features as well as our personal educational experiences, we developed ten learning modules with the goal to enhance dermatology knowledge for clerkship students, and emphasize the multidisciplinary nature and diverse relevance of dermatology in medicine and surgery.

**Table 1:** Outline of Beyond Skin Deep Modules

Case	Main Diagnosis	Skin Type	Overall Learning Objectives	Related Disciplines
1	Pemphigoid gestationis	II	Pregnancy dermatoses	Obstetrics
2	Lichen sclerosus	IV	Vulvar dermatoses	Gynecology
3	Trichotillomania	II	Approach to hair loss	Psychiatry & Family Medicine
4	Ocular rosacea	III	Approach to red eye	Ophthalmology
5	Cutaneous SCC	II	Management of cutaneous SCC	Family Medicine, Radiation Oncology & Pathology
6	Acne in PCOS	II	Management of acne	Gynecology & Family Medicine
7	Venous Ulcer	V	Approach to cutaneous ulcers	General Internal Medicine & Vascular Surgery
8	DRESS	VI	Approach to erythroderma	Emergency Medicine & Family Medicine
9	Acral Lentiginous Melanoma (T3a)	IV	Staging/management of melanoma	General Surgery, Plastic Surgery & Pathology
10	Primary syphilis	III	Cutaneous manifestation of syphilis	Family Medicine, Infectious Diseases & Public Health

*SCC* squamous cell carcinoma, *DRESS* drug reaction with eosinophilia and systemic symptoms, *PCOS* polycystic ovarian syndrome

The educational structure of the ten modules was case-based, stepwise, and composed of 8–10 multiple choice questions (MCQ) with detailed explanations, five learning objectives, and five take-home messages. The MCQ were developed by our team to cover relevant learning objectives important for each case. Each module took approximately 20–40 min to complete depending on prior familiarity of the topic. When the students are provided with the link to the website, they will select one of the 10 cases and follow the instruction given afterwards. The clerkship student assumes the role of a first-year resident in various disciplines to help prepare for the increased responsibility in patient care that comes with residency. In each case, the student is presented a complete patient profile followed by patient interview and clinical photos. The eight to 10 questions per case provide students with a comprehensive overview of relevant topics including rash morphology, differential diagnoses, clinical decision-making, investigations, diagnosis, treatment considerations, and prognosis. The case epilogue emphasizes long-term disease outcomes and follow-up considerations, while the take-home messages provide students with key information for future practice when encountering a similar dermatological presentation. The goal of the modules is to simulate clinical experience in dermatology for clerkship students.

The module content emphasizes the multidisciplinary care in dermatology and incorporates patients with different socioeconomic status and skin color, to encompass clinical presentations emphasizing the diverse population that clerkship students will encounter when treating skin conditions. For example, in the modules, clinical photos of Skin Color Type IV, V, and VI were included. In addition, the virtual patients in the case have diverse social backgrounds addressing the issues of homelessness, immigrant health, etc. Ten relevant topics were selected to reinforce curricular materials (e.g., skin cancer) as well as introduce new content. These topics overlapped with 14 disciplines in addition to dermatology (Table 1). Students are free to explore dermatology content relevant to their specific medical interests. For example, a case on pemphigoid gestationis can teach the intersection of dermatology with an obstetrical emergency. A case on ocular rosacea highlights the extensive crossover between ophthalmology and dermatology, as well as covering a topic not typically included in medical school curricula. Furthermore, a case on trichotillomania can emphasize how dermatology and psychiatry converge, and how both are important in the management of this difficult to diagnose condition. The modules were made available to students online in January 2021 (www.ualbertadermatology.ca/e-learning-modules) (password: dermatology).

### Implementation

We distributed the survey via email to clerkship students in January 2021 in two Canadian medical schools and promoted this study using appropriate class Facebook groups. Clerkship students were in the middle of clerkship training at that time. Total number of clerkship students reached was 305. Participants voluntarily completed the surveys anonymously and results were collected via Qualtrics, a survey management tool.

### Evaluation (i.e., Measuring outcomes)

Our study is a mixed methods study that aims to gather both quantitative and qualitative data. Participants who completed the pre-intervention (needs assessment) survey were invited to complete the post-intervention survey, which was available until August 2021. The survey was composed of five-point Likert scale ratings (1 = strongly disagree, 2 = disagree, 3 = neutral, 4 = agree, 5 = strongly agree) to elucidate feedback on the format as well as the impact of these modules on students’ dermatology knowledge and understanding of multidisciplinary care in dermatology. There are some relevant open-ended questions in the survey to gather qualitative feedback from students.

### Data analysis

An average score out of five with standard deviation was calculated for each statement. Qualitative feedback was also gathered regarding the content, format, ways to incorporate the modules into the curriculum and the multidisciplinary nature of the modules. Thematic analysis of qualitative data was used to identify major relevant themes [11]. All qualitative statements collected from the survey were gathered. Two authors reviewed the statements independently and identified major themes; consensus was reached after discussion. Analysis of the results aimed to help guide the development of future modules and identify opportunities for continuous improvement of module framework or content.

### Ethics

This study was approved by the university ethics boards (ethics ID: Pro00103200).

## Results

### Needs assessment survey

Eighty-nine out of 305 clerkship students (29.2%) from two Canadian universities completed the needs assessment survey. Participants reported interest in 24 different disciplines as their future career indicated by their desires to apply for Canadian residency matching. The top five disciplines were family medicine (*n* = 63; 70.8% of all participants), internal medicine (*n* = 37; 41.6%), pediatrics (*n* = 23; 25.8%), emergency medicine (*n* = 19; 21.3%), and dermatology (*n* = 15; 16.9%). Apart from curricular materials, surveyed clerkship students reported that they often do not use any additional educational resource that is specifically designed for them, and 12.4% of them do not use any additional resource at all to help them study dermatology.

Regarding perception of their dermatology curriculum, 17.1% of participants believed that the one-week pre-clerkship dermatology education they received is sufficient (Table 2). A minority (10.2%) of clerkship students felt comfortable managing patients with skin conditions in clinical settings. The majority of students agreed that they would encounter patients with skin conditions after finishing the residency of their desired specialty (89.6%) and 69.3% consider dermatology to be a specialty that often involves multidisciplinary care. For all statements in Table 2, the average Likert scale ratings are significantly different (*p* < 0.001) when compared with the value of 3 (neutral).

**Table 2:** Needs assessment survey of opinions regarding pre-clerkship dermatology curriculum

Statement	1	2	3	4	5	Mean (SD)	*P* value*
I feel the pre-clerkship dermatology education I received is sufficient	12.5%	44.3%	26.1%	14.8%	2.3%	2.50 (0.97)	< .001
I feel comfortable seeing patients with skin conditions in clinical settings	22.7%	40.9%	26.1%	6.8%	3.4%	2.27 (1.00)	< .001
After finishing the residency of my desired specialty, I will likely encounter patients with skin conditions	0.0%	4.6%	6.8%	25.0%	64.6%	4.48 (0.81)	< 0.001
I would describe dermatology as a specialty that often involves multidisciplinary care	0.0%	10.2%	20.5%	46.6%	22.7%	3.82 (0.90)	< 0.001

1 = strongly disagree, 2 = disagree, 3 = neutral, 4 = agree, 5 = strongly agree; SD = standard deviation

^*^ One sample t-test of data compared to 3 (neutral)

### Post-educational module intervention survey

**Fig. 1:**
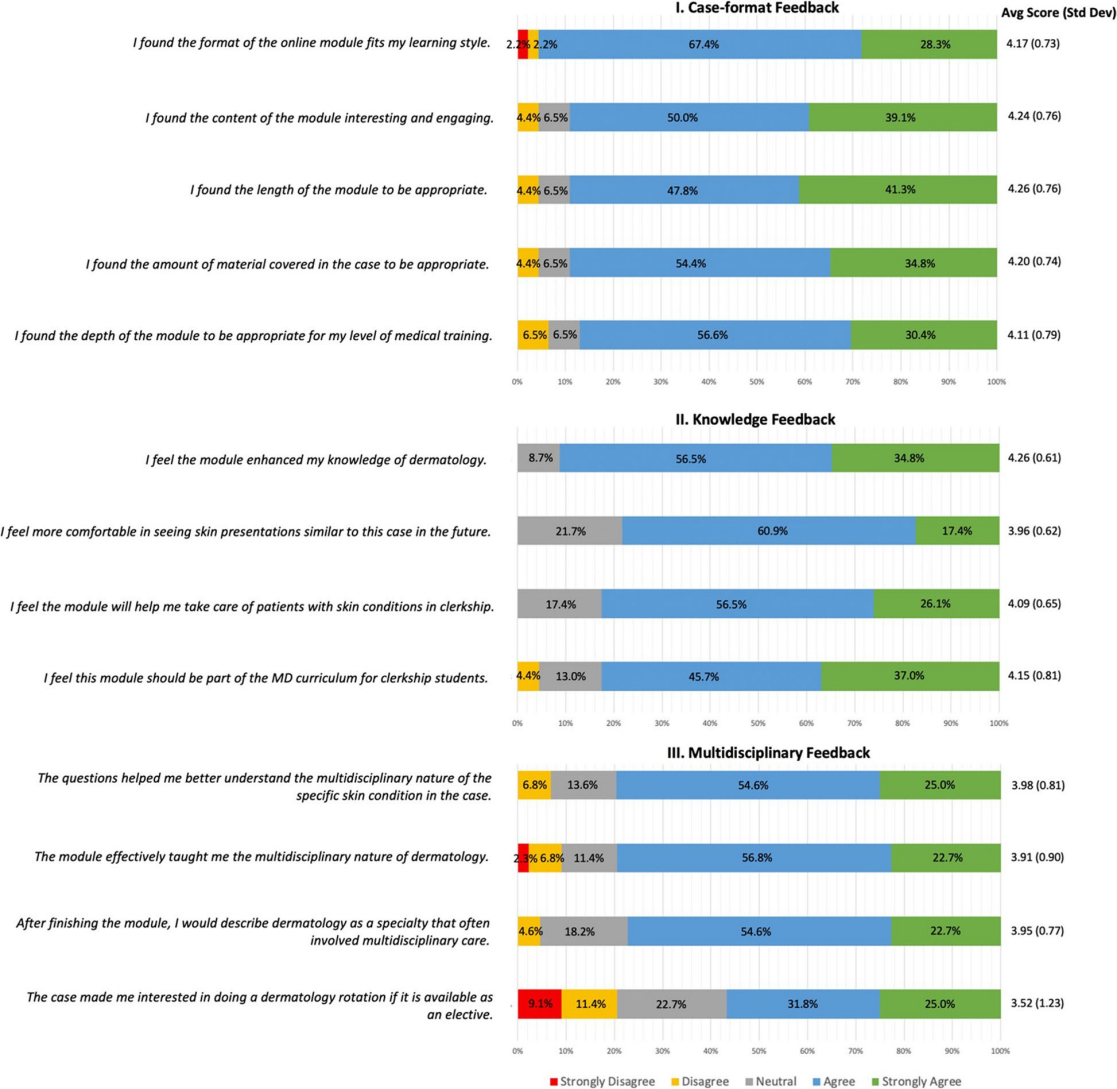
Feedback regarding case-format, knowledge, and multidisciplinary nature for modules

There were more than half (46 students; 51.7%) among the 89 who completed the pre-intervention survey, who also accomplished the modules and post-intervention survey afterwards. Students reported positive feedback regarding the format of the module (95.7% agreement), followed by the amount of material covered (89.2%), content (89.1%), length (89.1%), and the depth of knowledge covered in the cases (87.0%) (Fig. 1).

The majority (91.3%) of students felt the module enhanced their dermatology knowledge (Fig. 1). In comparison with 10.2% from the pre-intervention survey, 78.3% felt more comfortable managing skin presentations similar to the module cases. The vast majority of students believed that the modules would help them take care of patients with skin conditions in clerkship (82.6%) and that the modules should be part of the MD clerkship curriculum (82.7%).

For the multidisciplinary care component, 79.6% of students agreed that the modules helped with understanding the multidisciplinary nature of dermatology (Fig. 1), and 79.5% believed that the modules were effective at achieving that goal. Compared with 69.3% pre-intervention which is the majority of the students, 77.3% of students felt that dermatology is a specialty that often involves multidisciplinary care in dermatology. The difference is not statistically significant.

### Narrative feedback

Both positive and constructive feedback were received regarding module content and format, ways to incorporate the modules into the curriculum, and the multidisciplinary nature of the modules. Using thematic analysis of qualitative data (from open-ended post-module survey questions), five themes emerged that were identified after discussion and consensus among the two independent reviewers (Table 3) [12]. The themes were 1. Feasibility of module format, 2. Need of more visual aids, 3. Incorporation of modules (pre-clerkship and clerkship), 4. Benefits of multidisciplinary care, and 5. Indications of dermatology referral. Each theme was supported by an average of 22 statements (standard deviation of 5.7) with examples shown in Table 3. In summary, participants believed that the case-based style of the modules was engaging, easy to follow, and similar to real-life clinical cases. The images, description of rash morphology, and answer explanations were described as helpful; and the cases covered important and relevant materials that were not taught in the current curriculum. The constructive feedback included the need for videos or more clinical images to aid learn dermatology. Several ideas were provided regarding ways to incorporate the learning modules into the educational curriculum either during pre-clerkship or clerkship years as shown in Table 3. Most students believed that the modules could be incorporated into the dermatology week during pre-clerkship to have early exposure. Additionally, other possible time includes during other non-dermatology rotations, before exams, during the period of transition to clerkship, or the study period prior to the Canadian medical licensing exam.

**Table 3:** Qualitative feedback

Themes	Illustrative Quotes
Feasibility of module format	• Easy to follow, content is clear and concise • Pregnancy rashes were not adequately covered in medical school, and this supplements that weakness well • Dermatology pictures difficult to interpret and appreciated the description of morphology given in the case •Questions asked were diverse, and answer explanations were thorough and direct • The cases with the images, descriptions, and questions throughout felt like a real clinical case • Case-based style of the module was very fun, and the questions kept me engaged
Need for more visual aids	• Having pictures for each condition in follow up questions would be nice • More pictures and perhaps even video content would make the content even more engaging and interactive • More diagrams throughout might help solidify concepts • When certain signs and tests like pathology were mentioned, I would really love to see what they look like
Incorporation of modules (pre-clerkship)	• The modules can be incorporated into the dermatology week in pre-clerkship • Having derm as part of each block would be helpful • Before finals/derm exam, and again during the transition to clerkship • The one week of derm we had was super packed and content dense. I would like the modules to be optional • Some of the conditions seen are not taught in dermatology block and may be slightly above pre-clerk level
Incorporation of modules (clerkship)	• A module-based two-week mandatory dermatology rotation would help immensely with clerkship • It would be valuable to have 2–3 cases that are required to be completed for different non-derm rotations • Optional module for students to complete during their dermatology or family medicine rotations • For review in 4th year as part of recommended schedule for study for the licensing exam
Benefits of multidisciplinary care	• Systemic diseases can be diagnosed by a dermatological manifestation and will require referral and follow-up with other specialists in both the hospital and community settings • These cases showed how important knowledge of skin conditions is no matter what specialty you choose • I didn't know dermatology-psych clinic existed. This demonstrates how broad dermatology is • This module will help me to be a more well-rounded physician in the future in Rural Family Medicine • With outpatient exposure (mostly moles and psoriasis), from the cases, I learned how someone with a drug reaction could present and how different services would be consulted to manage the patient
Indication of dermatology referral	• It's not clear when you will involve other disciplines for the cases, for example, when should a GP refer? When should ophthalmology be involved? • I recognize the role of referral to different medical specialty as a form of multidisciplinary practice. I believe this isn't more or less evident in dermatology than it is in other areas of medicine

Students understood that diseases with skin manifestations require referral and follow-up with specialists beyond dermatology. For example, some students learned about the existence of dermatology-psychiatry clinics for primary psychodermatological conditions. Other students reported that the modules filled the learning gap of knowing how to involve the dermatology service in the inpatient setting. However, some students reported that the modules were not clear about when other disciplines should be involved for skin conditions. Students wanted further clarity of how multidisciplinary care in dermatology is different from multidisciplinary care in other specialities.

## Discussion

Skin disease is pervasive across fields of medicine and surgery, with all physicians likely to encounter dermatologic conditions in their career [11]. However, a limited number of dermatology electives (a limited number of weeks allowed for students to decide and spend time on different specialties) for medical students adds to the challenge of providing comprehensive education for non-dermatologist trainees [7]. Given the prevalence of skin disease, we also hope students with career interests in other specialties can learn more about the relevance of dermatology to their future career. Our goal is to further equip medical students to provide more comprehensive patient care when they move on to their next stage of medical training as non-dermatology residents and practising physicians in the future.

Due to limited clinical time and teaching resources in dermatology, our study demonstrated that case-based online modules are a platform to support medical education in dermatology, given the ease of accessibility and ability for students to select modules relevant to their learning goals and future careers. Our study presents an option of supplementing dermatology teaching for medical educators to consider, as the published, evidence-based literature for alternatives of improving dermatology education for medical students is limited [13].

The findings from the needs assessment survey emphasize that there is a substantial percentage of students who feel unprepared to manage patients with skin conditions during clerkship. This is consistent with previously published data [14–16]. This survey also showed that many students felt their one-week pre-clerkship dermatology education is insufficient. This is consistent with the perspective from Canadian undergraduate dermatology directors [6]. The educational resources designed specifically for medical students were rarely used based on the survey results, and it suggests that potential changes in curricula may aid in increasing students’ competency in dermatology. This emphasizes the need and role of our educational intervention. Specifically, there are several key aspects of our case-based educational intervention. First, the intervention is specifically designed for clerkship students. Also, students who are interested in specialties other than dermatology have the freedom of picking the modules based on their interests to achieve the goal of individualized learning. Next, students take on the role of a first-year resident to manage patients and learn how to navigate the multi-disciplinary collaboration in dermatology. In addition, the majority of Canadian medical schools have limited dermatology teaching or curricular time specific for clerkship students [6]. Important dermatologic conditions relevant to other non-dermatologic disciplines are not covered. The designed modules try to close this knowledge gap by educating on key dermatologic disorders relevant to specific medical specialties. From a boarder perspective, we hope the format of online case-based modules with a focus on multidisciplinary care can be used more globally to help medical students learn dermatology.

Our study showed that case-based online modules with MCQ, learning objectives, and take-home messages are an educational tool that fits learning style for students who completed the modules. The online platform is easily accessible and sharable, and it may solve the challenge of a lack of dermatology departments to facilitate incorporation of dermatology curricula [11]. The positive feedback for the modules is consistent with previously published data suggesting an online curriculum is highly acceptable to learners [17]. The qualitative feedback emphasized that students were interested in looking at clinical images; therefore having more clinical photos would be helpful in the development of future online educational dermatology modules. Previously identified challenges of incorporating dermatology into medical school curricula include limited time in the schedule and difficulty incorporating substantial dermatologic education into already established courses [13]. The qualitative feedback also suggested timeframes to introduce multidisciplinary dermatology educational resources apart from the designated dermatology week in clerkship. This includes non-dermatology blocks in pre-clerkship, the transition period to clerkship, non-dermatology rotations during clerkship, and pre-licensing exam study period. Overall, our results suggest that students are open to having longitudinal exposure to dermatology education, especially considering that the one week of dermatology in pre-clerkship is already perceived as very dense. In addition, further detailed evaluation of current MD curricula and feedback from students may help educators to properly fit in more curricular time for dermatology.

Following completion of the modules, the percentage of students who felt comfortable managing skin conditions increased 7.7 times from 10.2% to 78.3%. Additionally, 82.6% of students believed they could better take care of patients with skin conditions in clerkship following module completion while donning of the role of a first-year resident and going through the steps of describing rash morphology, generating differential diagnoses, and making clinical decisions of investigations and treatment. Overall, online education is a promising avenue of delivering and supplementing medical student dermatology education. However, it is important to recognize that only 52% of students completed the post-intervention survey which may contribute to selection bias. Although the intervention showed positive outcome, the students who did not complete the post-intervention survey may have different perspectives. Strategies to achieve higher post-intervention survey completion rate should be considered to evaluate the efficacy of the intervention more comprehensively.

79.6% of students who completed the modules agreed that the latter helped with understanding the multidisciplinary nature of dermatological cases, and the average Likert score is 3.98 ± 0.81 which is statistically significantly higher than the neutral value of 3. The percentage of students who described dermatology as a specialty that often involves multidisciplinary care increased from 69.3% to 77.3% after completing the modules. The difference is not statically significant, and it might be due to selection bias that students who participated in the study had a better understanding of the multidisciplinary care in dermatology and thus were interested in participating in this study. In addition, in future studies, it is important to explore students’ understanding of multidisciplinary nature of dermatology via focus groups.

As pointed out in the qualitative feedback, for similar initiatives to help medical students understand the multidisciplinary nature of dermatology, it is important to explain when to involve other disciplines for skin conditions, as well as further emphasize that dermatology often overlaps with many medical and surgical disciplines. Compared with high agreements with other statements, less students (56.8%) are interested in doing a dermatology rotation. As skin conditions are broad, it is important to allow students to choose topics of interest that are relevant to their future careers, especially for those who are not interested in dermatology. Having all students rotating through dermatology during clerkship may not be of interest for all students, therefore finding ways to engage these students’ interest is crucial. The format of our online modules provides flexibility for students to become familiar with dermatology content through the lens of different specialities.

There are several limitations with the study. First, the overall rate (15%) of all students who completed the modules is low given this is a pilot study with all modules optional to students. As emphasized earlier, 52% of students completed the post-intervention survey which is also low. Possible barriers for students to complete the post-intervention survey include lack of time from the clerkship student perspective, the length of the modules, or the length of the survey. Most students who completed the modules were interested in specialties in which dermatology is part of their training, so the results may be possibly skewed in a positive direction. Another limitation of our study was a lack of a robust student dermatology knowledge assessment pre- and post-intervention as the knowledge presented in these 10 modules was broad. Given that students’ perception of ability to manage conditions is intrinsically subjective, it is difficult to measure this objectively. Future studies that score students’ knowledge acquisition on the subject matter pre- and post-module may provide more objective assessment. Post-module questions that test students on when to involve specialties beyond dermatology may elucidate knowledge regarding multidisciplinary involvement. 

Overall, with this pilot study, we offer insight into ways of providing medical education for students when clinical teaching time and resources are limited. Our approach could be adapted to teach other disciplines that are not traditionally included in the clerkship medical school curricula including pathology, medical genetics, radiation oncology, etc. In summary, the Beyond Skin Deep online modules represent an initial contribution that we hope will continue to help students approach, diagnose, and manage primary dermatologic disorders as well as skin manifestations of diseases entailing multidisciplinary care.

## Supplementary Information

**Additional file 1: Appendix 1.** Pre-Module Survey. **Appendix 2.** Post-Module Survey.

## Data Availability

All data generated or analyzed during this study are included in this published article [and its supplementary information files].
