# Supplementary material for: Beyond Skin Deep: case-based online modules to teach multidisciplinary care in dermatology among clerkship students
Source: BMC Med Educ. 2023 Feb 4;23:90. doi: 10.1186/s12909-023-04072-z (PMC9898927; doi:10.1186/s12909-023-04072-z)
Supplement: Supplementary file 1 — Additional file 1: Appendix 1. Pre-Module Survey. Appendix 2. Post-Module Survey. [file 12909_2023_4072_MOESM1_ESM.docx]

**APPENDICES**

**Appendix 1:**

**Pre-Module Survey**

| **Part 1/2: Demographics** | | | |
| --- | --- | --- | --- |
|  | | | |
| 1. Level of education achieved (select all that apply) | Associate degree (2 and 3 years of undergrad)  Bachelor’s degree  Master’s degree  PhD  Other __________ | | |
| 2. Year of medical class | Class of 2021  Class of 2022  Currently doing MBA or PhD | | |
| 3. Specialty that you would consider applying for CaRMS (select all that apply) | Dermatology  Family med  Internal med  Pediatrics  Psychiatry  Emergency med  Neurology  Pediatric neuro  Physical med & rehab  Public health | General surgery  Anesthesiology  OBGYN  Orthopedics  Urology  ENT  Ophthalmology  Plastic surgery  Neurosurgery  Cardiac surgery  Vascular surgery | Radiology  Nuclear med  Medical genetics  Pathology  Medical microbiology |

| **Part 2/2: Background** | |  | | | | |
| --- | --- | --- | --- | --- | --- | --- |
|  | |  | | | | |
| 1. Apart from lecture notes, what resources have you used to study dermatology? | None  Toronto Notes  Google and read what are available  Dermatology specific textbooks (e.g., Bolognia)  UpToDate  Online modules (e.g., AAD modules)  Online video (e.g., OnlineMedEd, Osmosis)  DermNet NZ  Published paper(s)  Other __________ | | | | | |
|  | | *Strongly*  *disagree* | *Disagree* | *Neutral* | *Agree* | *Strongly*  *agree* |
| 2. I feel the pre-clerkship dermatology education I received is sufficient. | | 1 | 2 | 3 | 4 | 5 |
| 3. I feel comfortable seeing patients with skin conditions in clinical settings. | | 1 | 2 | 3 | 4 | 5 |
| 4. After finishing the residency of my desired specialty, I will likely encounter patients with skin conditions. | | 1 | 2 | 3 | 4 | 5 |
| 5. I would describe dermatology as a specialty that often involves multidisciplinary care. | | 1 | 2 | 3 | 4 | 5 |

**Appendix 2:**

**Post-Module Survey**

| **Part 1/3: Case-specific Feedback** |  | | | | |
| --- | --- | --- | --- | --- | --- |
|  | *Strongly*  *disagree* | *Disagree* | *Neutral* | *Agree* | *Strongly*  *agree* |
| 1. I found the format of online module fits my learning style. | 1 | 2 | 3 | 4 | 5 |
| 2. I found the content of the module interesting and engaging. | 1 | 2 | 3 | 4 | 5 |
| 2. I found the length of the module to be appropriate. | 1 | 2 | 3 | 4 | 5 |
| 3. I found the amount of material covered in the case to be appropriate. | 1 | 2 | 3 | 4 | 5 |
| 4. I found the depth of the module to be appropriate for my level of medical training. | 1 | 2 | 3 | 4 | 5 |
| 5. Do you have any additional comments about the module (e.g., things you like/dislike)? | _________________________________ | | | | |
|  |  | | | | |
| **Part 2/3: Knowledge Feedback** |  | | | | |
|  | *Strongly*  *disagree* | *Disagree* | *Neutral* | *Agree* | *Strongly*  *agree* |
| 1. I feel the module enhanced my knowledge on dermatology. | 1 | 2 | 3 | 4 | 5 |
| 2. I feel more comfortable in seeing skin presentations similar to this case in the future. | 1 | 2 | 3 | 4 | 5 |
| 3. I feel the module will help me take care of patients with skin conditions in clerkship. | 1 | 2 | 3 | 4 | 5 |
| 4. I feel this module should be part of the MD curriculum for clerkship students. | 1 | 2 | 3 | 4 | 5 |
| 5. How can the module be best incorporated into the MD curriculum? | _________________________________ | | | | |
| 6. How can the module be improved to boost your knowledge of treating skin conditions? | _________________________________ | | | | |
|  |  | | | | |
| **Part 3/3: Multidisciplinary Feedback** |  | | | | |
|  | *Strongly*  *disagree* | *Disagree* | *Neutral* | *Agree* | *Strongly*  *agree* |
| 1. The questions helped me better understand the multidisciplinary nature of the specific skin condition in the case. | 1 | 2 | 3 | 4 | 5 |
| 2. The module was effective in teaching me the multidisciplinary nature of dermatology. | 1 | 2 | 3 | 4 | 5 |
| 3. After finishing the module, I would describe dermatology as a specialty that often involves multidisciplinary care. | 1 | 2 | 3 | 4 | 5 |
| 4. The case made me interested in doing a dermatology rotation if it is available as a selective. | 1 | 2 | 3 | 4 | 5 |
| 5. How has completing the module changed your view on the multidisciplinary nature of dermatology and impacted your interest in the field of dermatology? | _________________________________ | | | | |
